# Tribbles expression in cumulus cells is related to oocyte maturation and fatty acid metabolism

**DOI:** 10.1186/1757-2215-7-44

**Published:** 2014-04-26

**Authors:** Daphné Brisard, Franck Chesnel, Sébastien Elis, Alice Desmarchais, Laura Sánchez-Lazo, Manon Chasles, Virginie Maillard, Svetlana Uzbekova

**Affiliations:** 1 INRA, UMR 85 Physiologie de la Reproduction et des Comportements, Nouzilly 37380, France; 2 CNRS UMR7247, Nouzilly 37380, France; 3 Université François Rabelais de Tours, Tours 37000, France; 4 IFCE, Nouzilly 37380, France; 5 CNRS, Université de Rennes 1, UMR6290, Rennes 35000, France

**Keywords:** *Tribbles*, Oocyte maturation, Cumulus cells, Fatty acid metabolism

## Abstract

**Background:**

In mammals, the *Tribbles* family includes widely expressed serine-threonine kinase-like proteins (TRIB1, TRIB2 and TRIB3) that are involved in multiple biological processes including cell proliferation and fatty acid (FA) metabolism. Our recent studies highlighted the importance of FA metabolism in cumulus cells (CC) during oocyte maturation in vertebrates and reported a higher *TRIB1* expression in CC surrounding in vivo mature oocytes as compared to immature ooocytes in mice and cows. The objective was to investigate *Tribbles* expression patterns in bovine CC during in vitro maturation (IVM) and to examine their roles in the cumulus-oocyte complex.

**Methods:**

*Tribbles* gene expression was analyzed in bovine and murine CC using quantitative RT-PCR. Proteins were detected using Western blot and intracellular localization was assessed by immunofluorescence. Bovine COCs were treated with etomoxir, an inhibitor of FA oxidation (FAO) which blocks CPT1 activity, during 6 h and 18 h IVM. Oocyte meiotic stage was assessed and expression of *Tribbles* and lipid metabolism genes was quantified in CC.

**Results and discussion:**

*TRIB1* and *TRIB3* were more strongly expressed whereas *TRIB2* was less expressed in CC surrounding the oocytes from preovulatory follicles than in CC of immature ones. In CC, Tribbles were located in the cytoplasm and nucleus; in mitotic cells TRIB2 and TRIB3 were detected in the spindle. In the oocyte, Tribbles proteins were detected in the ooplasm; also TRIB2 and TRIB3 were more accumulated in the germinal vesicle. In bovine CC, expression of *TRIB1* and *TRIB3* was transiently increased at a time preceding oocyte meiosis resumption in vitro. Treatment with etomoxir (150 μM) during IVM resulted in a significant reduction of oocyte maturation rate and decreased MAPK3/1 phosphorylation in the oocytes. In CC, 18 h IVM of etomoxir treatment significantly increased expression of *TRIB1, TRIB3, CPTA1* (enzyme regulating FA entry in mitochondria for FAO) and *CD36* (thrombospondin receptor involved in FA transport). Under the same conditions, expression of *TRIB2 and ACACA* (Acetyl coenzyme A carboxylase involved in FA synthesis) decreased in CC.

All considered, *Tribbles* family members may be involved in cell proliferation and in FAO signaling in CC and participate in oocyte meiotic resumption regulation.

## Introduction

*Tribbles* genes, first identified in *Drosophila melanogaster*[1], have three homologues in mammals: *TRIB1*, *TRIB2* and *TRIB3*. All *Tribbles* family members are serine-threonine kinase-like proteins which present three motifs: 1) a divergent kinase region with undetermined catalytic activity corresponding to the trib domain, 2) a COP1 site allowing key proteins to be targeted to the proteasome for degradation and 3) a MEK1 binding site that modulates Mitogen Activated Protein Kinase (MAPK) activity. *Tribbles* genes are well conserved throughout the metazoan lineage [2]. Among the human Tribbles proteins, TRIB1 and TRIB2 share 71% homology, TRIB1 and TRIB3, 53%, and TRIB2 and TRIB3 share 54% homology [3]. Tribbles exert multiple functions and their expression is tissue-dependent. Tribbles proteins have been described in numerous processes such as cell division and migration, tissue homeostasis, inflammation or carcinogenesis in different tissues [4]. Tribbles proteins not only act as scaffold proteins but exert additional tissue-specific functions; notably TRIB1 and TRIB3 were shown to be involved in lipid homeostasis [5]. TRIB1 has been associated with deregulated triglycerides and cholesterol levels in plasma in humans [6] and was shown to regulate lipogenesis in mice hepatic cells [7]. It was demonstrated that a lack of amino acids or glucose induced an increase in TRIB3 protein level (reversible if fresh nutrients were added) making it an indicator for nutrient starvation [8]. Finally, it was shown that *TRIB3* could prevent fat accumulation in adipocytes [9].

Tribbles family proteins have never been studied in the ovarian follicles of mammals and their function in ovarian cells is still unknown. Interestingly, Trib1-deficient female mice and Drosophila in adulthood are both infertile (unpublished data cited by Yamamoto et al. [10]). Our recent study dealing with follicular cells surrounding the oocytes before and after meiotic maturation in different species has reported *TRIB1* among the genes upregulated in mature follicles of three tetrapods: cow, mouse and *Xenopus* inferring its involvement in granulosa/cumulus cell functions during oocyte maturation [11]. According to transcriptome analysis, in vivo *TRIB2* was down-regulated during the periovulatory period in bovine granulosa cells [12] and in CC at 6 h following LH surge [13]. These observations hypothesized that Tribbles in follicular cells may have a role during the final stages of folliculogenesis and oocyte maturation. It is well established that MAPKs along with energy metabolism in follicular cells, are essential for proper maturation of the enclosed oocyte and for subsequent fertilization [14-16]. Indeed the oocyte needs energy to perform maturation including meiosis resumption from prophase-I to the metaphase-II stage. This developmental step involves different signaling pathways within the cumulus-oocyte complex (COC) which is held by MAPK ERK1/2 activation in the oocyte [14]. Fatty acids (FA), as well as glucose derivatives and amino acids, are the energy source for the oocyte and are transported to the oocyte *via* surrounding cumulus cells (CC) [17,18]. The importance of FA metabolism in CC for *in vivo* oocyte maturation in mammals such as the mouse and the cow was also highlighted by functional genomics studies [11]. In that latter study, we showed that numerous genes involved in FA metabolism were differentially expressed between immature and preovulatory CC in mice and cows, including *TRIB1*.

Moreover we have also observed that CC may regulate oocyte lipid utilization and metabolism during *in vitro* maturation (IVM) in bovine [19]. In the bovine oocyte, triglycerides are the most abundant intracellular lipids, and palmitic acid is the most representative FA [20]. Energy from FA is released by mitochondrial fatty acid β-oxidation (FAO); the carnitine palmitoyltransferases (CPT) are the enzymes underlying the oxidative reaction [21]. CPT1 allows the FA entry in the mitochondria by catalizing the transfer of the acyl group of long-chain fatty acid-CoA conjugates onto carnitine. *In vivo*, malonyl-CoA which is synthesized from acetyl-CoA through the acetyl-CoA carboxylase (ACC) can inhibit CPT1 [22]. In mouse adipose tissue, TRIB3 inhibited the ACC enzymatic activity by enhancing its degradation and stimulated FAO [23]. Recent studies reported that FAO in mouse COCs is very important for oocyte meiotic resumption, maturation progress and early embryo development [24-26]. Thus, *in vitro* supplementation of mouse follicles with CPT1 activator l-carnitine significantly increased beta-oxidation and markedly improved both fertilization rate and blastocyst development [27], whereas administration of CPT1 inhibitor etomoxir during oocyte maturation impaired embryo development [28]. Moreover, in the presence of FAO inhibitor etomoxir, *in vitro* meiotic maturation was delayed in mice, porcine and bovine COCs and this delay was accompanied by alteration of lipid metabolism-related genes including those encoding CPTs, in the oocyte and in CC [29]. *Tribbles* genes are involved in lipid metabolism in different cell types and participate in the regulation of AKT/protein kinase B [30], MAPK [31], and SMAD [32] signaling pathways. In bovine CC and oocytes, these signaling pathways are also involved in progression of oocyte maturation [33,34].

In this context, our objective was to characterize the expression of *Tribbles* genes in CC during oocyte maturation. We also explored the possible involvement of *Tribbles* in lipid metabolism in these cells.

## Methods

### Ethics

All procedures on animals were approved by the Agricultural and Scientific Research Government Committee (approval number A37801) in accordance with the guidelines for the Care and Use of Agricultural Animals in Agricultural Research and Teaching.

### Chemicals

All chemicals were purchased from Sigma (Saint-Quentin Fallavier, France) unless otherwise stated.

### Microarray data analysis

Microarray GEO datasets reported using murine CC (series GSE36604) and bovine CC (series GSE36605) published in Charlier et al. [11] were used. Mean expression values of *Trib1, Trib2* and *Trib3* genes before and after maturation and correspondent statistics were extracted from each microarray hybridization set (n = 6 per condition).

### Cumulus cell collection

In mice, COCs were obtained from 8-week old B6CBAF1/J mice (Charles River Laboratories, Larbresle Cedex, France; 5–10 animals per group). Immature COCs were isolated from non-primed mice, and *in vivo* mature COCs were obtained from mice primed in the first 48 h with a single peritoneal injection of PMSG (5 IU) followed at 16 h with hCG (5 IU, Schering-Plough, Courbevoie, France), as previously described [11].

Bovine immature COCs were obtained from slaughterhouse ovaries of cows of different races as described below whereas *in vivo* mature COCs were recovered by ovum pick up after FSH/LH-based ovarian stimulation protocol from 5 non-lactating cows as previously described [35]. Immediately after COCs collection, CC were removed from the oocytes, pelleted and stored in 100 μL TriZol reagent (Invitrogen, Cergy Pontoise, France), snap-frozen in liquid nitrogen and kept for RNA extraction at −80°C.

### *In vitro* maturation (IVM) of bovine COCs

Follicles from 3 to 6 mm were punctured from slaughterhouse ovaries using an 18G needle linked to a vacuum pump and to a falcon tube of 50 mL. Liquid recovered from follicular puncture and containing immature COCs was transferred into TCM 199H (Tissue Cell Medium 199-Hepes supplemented with 0.05% BSA and 25 μg/mL gentamycin) and COCs were picked up under binocular loupe. Only COCs with multilayers of CC and morphologically compact were chosen. After washing 3 times with the TCM199H, COCs were subjected to IVM in serum-free TCM199-enriched medium (199EM) containing gonadotropins and growth factors as described elsewhere [34]. Groups of COCs were incubated in 500 μL of 199EM at 38.8°C with 5% CO_2_ and 20% O_2_ for 24 h in order to complete IVM. Groups of COCs were sampled before IVM (0 h) and at 3 h, 6 h, 18 h and 24 h IVM.

In order to analyze the effect of FA oxidation inhibition, etomoxir in aqueous solution (final concentration, 150 μM) was added in TCM 199EM before IVM and the same volume of H_2_O was added as a control. This etomoxir concentration was chosen according to data reported on bovine COCs showing an oocyte maturation delay [29]. After IVM, CC were mechanically removed from the oocyte by a series of pipette aspiration-rejection movements in TCM199H. CC were pelleted by centrifugation, 100 μL TRIzol® reagent was added to the pellet and tubes were snap-frozen in liquid nitrogen and kept at −80°C up to RNA extraction.

### Assessment of cell viability

Viability of COCs after *in vitro* culture in the presence of CPT1 inhibitor etomoxir was checked using Live/Dead Viability/Cytotoxicity Kit for mammalian cells (Molecular Probes; Invitrogen) according to supplier’s instructions. Briefly, *in vitro* cultured COCs were rinsed in warm PBS pH7.4 and then covered with 200 μL of 2 μM calcein AM and 4 μM EthD1 solution in PBS. After 15 min incubation at 38.8°C, cells were observed using an inverted fluorescent Axioplan Zeiss microscope with appropriate filter set (excitation 469 ± 17.5 nm, emission 525 ± 19.5 nm) allowing simultaneous detection of both calcein AM (green) and EthD1 (red). Live cells appear green and dead cells red. Color microphotographs were taken using 11.2 Color Mosaic and Spot Advanced 4.0.1 software and compared between control and treated conditions.

### Assessment of oocyte maturation rate

Oocytes were stripped from CC by repetitive aspiration-ejection using a Gilson pipette and then immediately fixed in 4% paraformaldehyde PBS solution for 30 min at room temperature. Fixed oocytes were incubated in Hoechst33342 (0.1% in PBS) for 10 min and mounted on a glass slide in Mowiol solution. Chromatin labeling was observed under an Axioplan Zeiss fluorescent microscope (Zeiss, Germany). The oocytes were considered mature if telophase-I or metaphase-II stages were seen. Maturation rate was calculated as a percentage of mature oocyte in a total number of live oocytes.

### Total RNA extraction

Total RNA was extracted from CC (from groups before or after IVM) using TRIzol reagent according to the manufacturer’s recommendations. RNA concentration was determined using a NanoDrop spectrophotometer (Nyxor Biotech, Paris, France).

### Reverse transcription

Before RT, RNA samples were treated with RQ1 DNase (Promega) for 15 min at 37°C and cDNA was then extended from 500 ng of total RNA for 1 h at 37°C using mouse Moloney Leukemia Virus Reverse transcriptase and a mix of oligo-(dT)_15_ (0.025 μg per reaction) and dNTPs (0.125 mM) according to the manufacturer’s protocol (Invitrogen). CC cDNAs were diluted to a concentration of 0.2 ng/μL.

### Real-time PCR analysis

Real-time quantitative PCR (RT-qPCR) reactions were run on a MyiQ™ Cycle device (Bio-Rad, Marnes la Coquette, France) using the SYBR Green Supermix (Bio-Rad), 5 μL of diluted cDNA (equivalent to 1 ng of converted RNA) and 125 nM of specific primers (Additional file 1: Table S1) in a total volume of 20 μL. After denaturation at 95°C for 3 min, samples were subjected to 40 cycles composed of a three-step protocol (95°C for 30 sec, 60°C for 30 sec and 72°C for 20 sec) followed by acquisition of a melting curve. The efficiency of the primers was deduced by performing PCR from serial dilutions of cDNA fragments obtained as a template for each gene. To confirm the specificity of the amplified fragments, PCR products were verified by single-peak melting curve and then run on a 2% Tris-borate agarose gel to verify the predicted size of the amplified fragments.

The relative mRNA expression level was calculated for each target gene as follows. The mean value from technical duplicates (SQ) was considered for each sample, and then normalized by the geometric SQ value of two internal control genes *RPL19* and *RPS9* for cow’s CC. In mice, *18S* and *rplp0* were used to normalize gene expression values in CC. Four to six independent CC samples were analyzed in each condition.

### CC in vitro culture

CC were detached from 120 immature COCs by repeated pipette aspiration-ejection movements. CC were then centrifuged and resuspended in TCM199 supplied with 10% fetal calf serum, IGF (10^−8^ M), 100 U/mL penicillin, 10 μg/mL streptomycin and 25 μg/mL amphotericin. The cell suspension was distributed in 8-well Millicell EZ slides (Fisher Scientific, Illkirch, France) (250 μL per well) and incubated in humidified atmosphere at 38.8°C with 5% CO_2_ and 20% O_2_ for 18 h, and then the culture medium was changed once more time 24 h later. After 60 h of culture, medium was aspirated, cells were rinsed with warm PBS (pH 7.4) and fixed in 4% paraformaldehyde solution in PBS for further immunofluorescence analysis.

### Immunofluorescence

Bovine and murine COCs or CC after in vitro culture were fixed during 30 min in 4% paraformaldehyde solution in PBS (pH 7.4). After fixation, cell permeabilization was performed in PBS/BSA 0.5%/Triton X100 0.5% for 15 min. Cells were then incubated in PBS/BSA 2% supplemented with 5% goat or horse serum for 1 h to block non-specific binding sites. A wash with PBS/BSA 0.1%/Tween 20 0.1% preceded the overnight incubation with the primary antibodies at 4°C. Goat antibodies against recombinant human (rh) TRIB1 (Santa Cruz Biotechnologies, Heidelberg, Germany), rabbit rh TRIB2 antibody and rabbit rh TRIB3 antibody (Sigma) were used at 1:100 dilution. Four 15-min washes preceded the incubation of the secondary donkey anti-goat or goat anti-rabbit Alexa488-coupled antibodies (Molecular Probes, Invitrogen) at room temperature for 2 h. Four PBS/BSA 0.1%/Tween 0.1% washes of 20 min were performed and 1 μg/mL of Hoechst was added in the last one. Lastly a drop of Mowiol® anti-fade solution was added to mount the slides and allow confocal microscopy analysis. Immunofluorescence was observed using a Zeiss confocal microscope LSM700 (Carl Zeiss Microscopy GmbH, Munich, Germany) using oil 40× and 63× objectives.

### Western blot analysis

In order to extract the proteins, frozen cells were subjected to 3 melting-freezing cycles (1 min at 30°C and 1 min in liquid nitrogen). Protein concentration was measured using BCA protein kit (Interchim). Before loading, concentrated reducing Laemmli buffer containing dithiothreitol (final concentration = 80 mM) was added and the samples were boiled for 8 min. Proteins were resolved on 8-20% gradient SDS-PAGE gel and transferred onto nitrocellulose membranes. Blots were saturated with 5% nonfat milk powder in Tris-buffered saline/0.1% Tween 20 (TBS-T) for 1 h at room temperature and probed with diluted antibodies overnight at 4°C. Dilutions were 1:800 for TRIB1 goat antibody, 1:500 for TRIB2 rabbit antibody, 1:1000 for TRIB3 rabbit antibody, 1:2000 for phosphoMAPK3/1 (Thr202 and tyr204 for MAPK3; Thr185 and Tyr187 for MAPK1), 1:1000 for total MAPK3/1 rabbit antibody (Santa Cruz Biotechnology, Inc, Heidelberg Germany) and 1:1000 for alpha-tubulin (TUBA) monoclonal antibody. After washing, membranes were incubated for 1 h at room temperature with Horseradish peroxidase (HRP) conjugated goat anti-rabbit or donkey anti-goat (Cell Signaling Technology, France) and HRP-conjugated goat anti-mouse IgG (LabVision, Fremont, CA) diluted 1:10,000 in TBS-T. A signal was produced by chemiluminescence ECL Plus kit (Amersham Bioscience, Orsay, France). The signal was detected and revealed by imaging (G:BOX Chemi, Syngene, UK). Four or five independent samples were quantified for each condition. The data are presented as a ratio of signal mean intensities in arbitrary units of either phosphorylated protein to total protein or protein of interest to TUBA.

### Statistical analysis

Statistical analysis were performed using StatView 5 (SAS Institut Inc, US) and GraphPad Prism, (San Diego, CA) software. One way ANOVA and Tukey’s post-hoc test were performed when several conditions were compared. Non-parametric Mann Whitney test was used to compare two groups. The difference was considered significant at p < 0.05; trend was considered if 0.5 < p < 0.1. For graphics, the relative expression values were presented as a ratio to one of the groups.

## Results

### Tribbles characterization in the cumulus-oocyte complex

**Figure 1:**
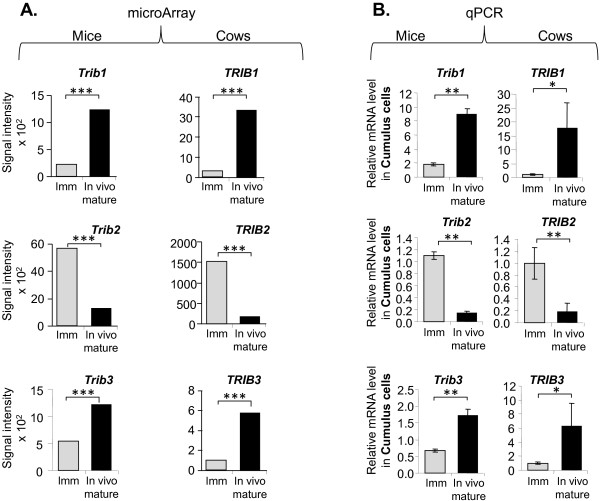
**Tribbles genes expression in murine and bovine cumulus cells (CC) surrounding immature and mature oocytes *in vivo*.** CC were recovered from either immature oocytes originating from small follicles (immature) or from metaphase-II oocytes from *in vivo* mature cumulus-oocyte complexes (COCs). **A.** Data from murine and bovine microarray analyses [11]. Histograms present mean expression values of six independent samples as detected by microarray hybridizations with correspondent p-values calculated using Fisher’s exact test and Benjamin-Hochberg correction). **B.** RT-qPCR analysis of Tribbles gene expression in CC. Graphs present mean +/−SEM of normalized expression values of five independent replicates. For statistics Mann–Whitney test was performed. Asterisks indicate significant differences (*p < 0.05, **p < 0.01; ***p < 0.001).

Analysis of microarrays datasets [11] revealed that *TRIB1*, *TRIB2* and *TRIB3* genes are expressed in CC of both mice and bovine. In mouse, as in cow, *TRIB1* and *TRIB2* were similarly regulated in CC surrounding immature *vs in vivo* mature pre-ovulatory (PO) oocytes. Expression of *TRIB1* was significantly increased after maturation whereas *TRIB2* decreased in both species; *TRIB3* transcript level was also increased in pre-ovulatory mature CC (5-fold in bovine and two-fold in mice) (Figure 1A). According to signal intensity of the microarray probes, *TRIB2* showed the highest and *TRIB3* the lowest expression level among the three *Tribbles* genes in both species. We performed real-time PCR quantification on independent CC samples from immature and mature oocytes of both species and confirmed these expression patterns in mice and cow (Figure 1B). Interestingly, in bovine CC *TRIB1* and *TRIB3* genes showed a higher variation between immature and pre-ovulatory CC than in mice.

According to microarray data, *Tribbles’* expression in bovine CC was higher after in vivo maturation compared to IVM. Thus, *TRIB1* was reduced 10.6-fold after IVM than in preovulatory CC, and *TRIB2* and *TRIB3* showed 2.2 and 1.5-fold decrease, respectively.

**Figure 2:**
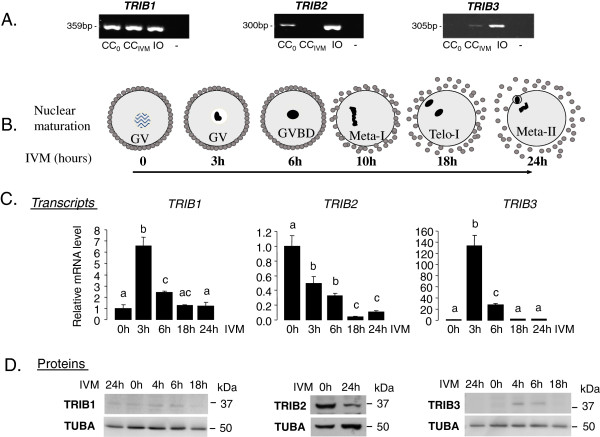
**Expression of Tribbles family members in bovine cumulus cells and oocytes in relation with meiotic progression in vitro. A.** RT-qPCR detection of *TRIB1, TRIB2* and *TRIB3* transcripts in bovine CC before (CC_0_) and after *in vitro* culture (CC_IVM_) and in immature oocytes (IO). Amplicon size is indicated on the left in base pairs (bp). **B.** Kinetics of meiotic progression in bovine oocyte during 24 h IVM as described [34,61]. GV – germinal vesicle, GVBD – GV breakdown; Meta-I –metaphase-I, Telo-I – telophase-I, Meta-II – metaphase-II. **C.** Kinetics of *TRIB1, TRIB2* and *TRIB3* gene expression in bovine CC during IVM. RT-qPCR was performed on CC recovered from the COCs at 0 h, 6 h, 18 h and 24 h of IVM. Graphs present means +/−SEM of normalized expression values of 4 independent CC samples. Different letters indicate a significant difference (ANOVA and Tukey’s post-hoc test, p < 0.05). **D.** Detection of TRIB1, TRIB2 and TRIB3 proteins in bovine COCs. Western blots using anti-Tribbles antibodies were performed on COCs collected at 0 h, 4 h, 6 h 18 h and 24 h IVM; TUBA was used as a loading control.

Detailed analysis of expression pattern of *Tribbles* genes was performed in bovine COCs. Using classic RT-PCR, the presence of *TRIB1, TRIB2* and *TRIB3* transcripts were verified in CC before and after IVM and in the immature oocyte (Figure 2A). Transcripts of all three *Tribbles* were detected in the oocytes, whereas in CC *TRIB2* was faintly detectable after IVM, in contrast toTRIB3 which was better detected in mature CC.

A time-course of *Tribbles* expression was followed in CC during IVM. The kinetics of nuclear maturation and cumulus modifications during IVM in our laboratory [34] is schematically presented in Figure 2B. The immature prophase oocyte with germinal vesicle (GV) is surrounded by compact layers of CC; after 3 h, IVM chromatin is condensed and GVBD occurs after 6 h. Oocytes reached metaphase-I near 10 h of IVM and further progress through the first meiotic division to metaphase-II stage at 18–24 h. Since 10 h IVM, cumulus became more expanded due to extracellular matrix formation, and gap-junctions between CC and oocytes are closed. The expression of *TRIB1, TRIB2* and *TRIB3* in CC was quantified by real time PCR (Figure 2C). Expression of *TRIB1* and *TRIB3* transiently increased after 3 h and 6 h of IVM compared to 18 h and 24 h (p < 0.05). At that time, their expression level was similar to that observed before IVM. *TRIB2* mRNA level decreased during IVM and was ten-fold lower than that before IVM (p < 0.05).

By Western blot analysis, by using TRIB1, TRIB2 and TRIB3 antibodies the proteins with molecular weights of approximately 37, 39 and 40 kDa, respectively, were detected in bovine COCs collected at different time points of IVM (Figure 2D) and the pattern of expression seems correlate with transcript variations.

### Intracellular localization of Tribbles proteins in CC and oocytes

**Figure 3:**
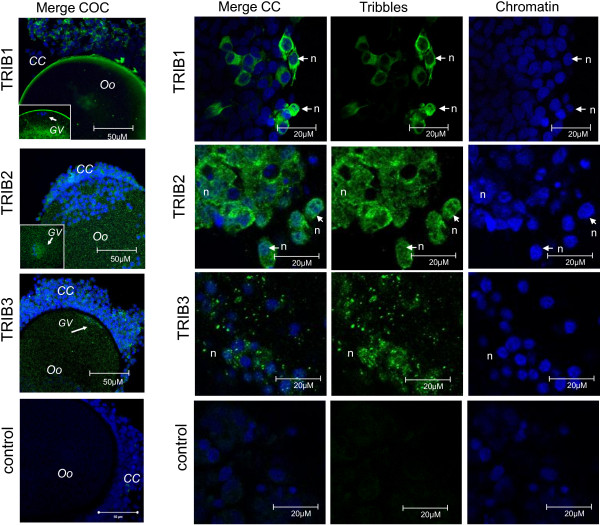
**Immunofluorescence detection of TRIB1, TRIB2 and TRIB3 proteins in bovine cumulus cells (CC) and the oocytes.** First column represents typical merged confocal images of Tribbles and Hoechst labeling of entire cumulus-oocyte complex (COC). Three right columns are images of CC. Antibodies against rh TRIB1, TRIB2 and TRIB3 proteins and Alexa488-coupled secondary antibodies were used to detect specific labeling. Rabbit IgG was used as negative control. Bars, 50 μM (first column) and 20 μM (others columns). Arrows point to the nucleus (n).

Intracellular localizations of Tribbles proteins were analyzed in bovine and in murine COCs using immunofluorescence performed *in toto* (Figure 3)*.* In bovine, TRIB1 protein was detected in some of the CC and it was located mainly to cytoplasm; TRIB2 and TRIB3 were visible in all CC, both in cytoplasmic and nuclear compartments. TRIB3 was detected as numerous spots inside the CC cytoplasm, and labeling differed between the cells. In bovine oocytes, TRIB1, TRIB2 and TRIB3 fluorescence was detected in the ooplasm. Interestingly, TRIB1 was mainly located closely to ooplasm membrane whereas TRIB2 and TRIB3 were more concentrated in GV closely associated to chromatin.

In mouse CC, TRIB2 was mostly detected in the cytoplasm whereas TRIB3 was more concentrated in the nucleus of CC (Additional file 2: Figure S1). Specific TRIB2 and TRIB3 labeling was also detected in mouse oocytes. Trib2 and Trib3 proteins were distributed throughout the ooplasm, but highly concentrated in GV.

**Figure 4:**
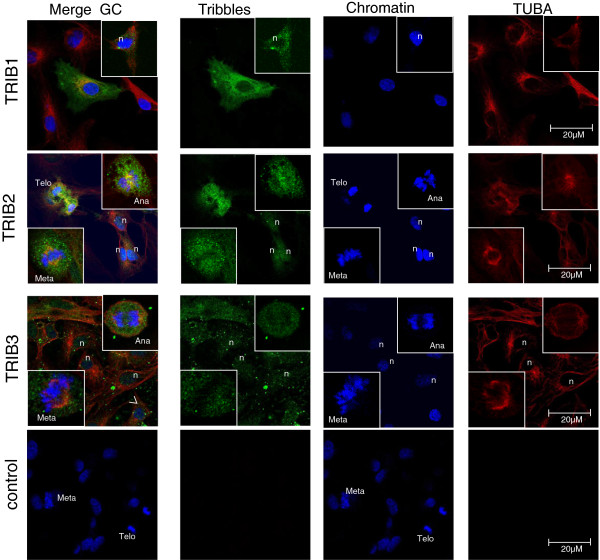
**Intracellular localization of TRIB1, TRIB2 and TRIB3 proteins in bovine cumulus cell cultured *in vitro*.** Cells were fixed after 60 h of culture and double immunofluorescence was performed using primary antibodies against TUBA (red) and either rh TRIB1, or TRIB2 or TRIB3 proteins (green). For the negative control, primary antibody was replaced by mice and rabbit IgG. Chromatin is labeled blue. Nucleus (n), Metaphase (Meta), anaphase (Ana) and telophase (Telo) are indicated in some of the cells.

Tribbles proteins were also detected in bovine CC monolayers after *in vitro* culture (Figure 4). Both nuclear and cytoplasmic localization were noted for TRIB1, TRIB2 and TRIB3 proteins. TRIB1 was detected in some of the CC and was concentrated in bright spots and around the nucleus, where it partially colocalized with alpha-tubulin. In mitotic cells, TRIB2 was distributed around the chromatin and concentrated closely to spindle in metaphase. In anaphase and telophase cells, TRIB2 was localized around the chromosomes and concentrated to the interzone where it partially colocalized with alpha-tubulin (TRIB2 panel insets, Figure 4). TRIB3 was also detected as numerous bright spots inside CC. In metaphase and telophase cells, TRIB3 was partially colocalized with alpha-tubulin to the spindle (TRIB3 panel insets, Figure 4).

### Effect of etomoxir on Tribbles gene expression in CC and on oocyte maturation

In order to test whether *Tribbles* expression is related to lipid metabolism in CC, the effect of etomoxir, an inhibitor of FAO that blocks CPT1 activity, was evaluated on bovine CC during IVM. Etomoxir was used at a final concentration of 150 μM which had been recently shown to reduce oocyte maturation rate in bovine COCs [29]. A test of viability was performed on treated and control COCs at 6 h and 18 h IVM and revealed that viability was not significantly affected compared to the control (Additional file 3: Figure S2).

**Figure 5:**
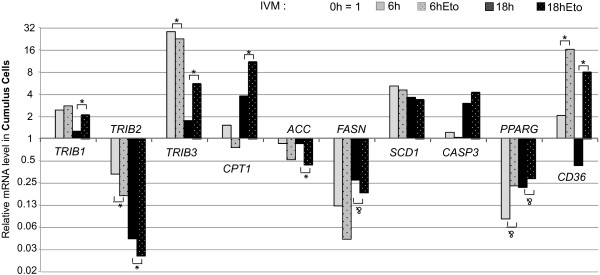
**Effect of etomoxir (150 μM) on expression level of *Tribbles *and lipid metabolism related genes in bovine CC after 6 h and 18 h of IVM as detected by real time PCR.** Bars are mean of normalized expression values (log2) of 4 independent replicates. 0 h IVM is considered as 1. Mann–Whitney test was performed to compare etomoxir–treated samples with correspondent controls. Asterisks mean significant difference with p < 0.05. Trend is denoted as & with 0.05 < p < 0.1.

Expression of *Tribbles* and other genes related to lipid metabolism (*ACACA, SCD1, PPARG, FASN, CPT1A, CD36)* were measured before and after 6 h or 18 h of IVM in the presence or absence of etomoxir (150 μM), using real time PCR (Figure 5). Expression of all studied genes in CC significantly varied in at least one time-point throughout 6 h-18 h IVM as compared to 0 h maturation (ANOVA, p < 0.05, statistics not shown). After 6 h IVM in the presence of etomoxir, expression of *TRIB2* and *TRIB3* was significantly lower (two-fold and 21% decrease, respectively, p < 0.05) and *CD36* expression was 16-fold higher as compared to control non-treated CC (p < 0.05). After 18 h IVM in the presence of etomoxir, expression of *TRIB1, TRIB3, CPTA1* and *CD36* in CC was significantly higher in the control condition (1.7-fold, 3.2-fold, 3.0-fold and 18-fold increase, respectively, p < 0.05). At the same time, significant decrease of *TRIB2* and *ACACA* expression levels (40% and 48% decrease, respectively) were induced in etomoxir treated CC compared to control CC.

**Figure 6:**
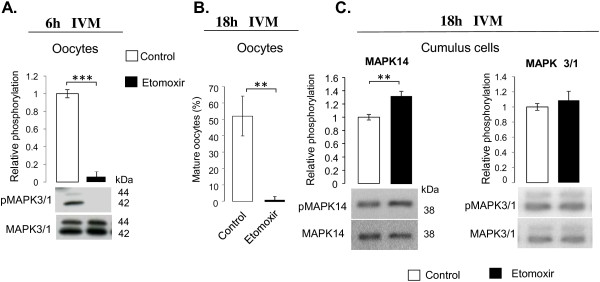
**Effect of etomoxir (150 μM) on oocyte maturation and signaling pathways. A.** Phosphorylation of MAPK3/1 in bovine oocytes after 6 h IVM **B.** Oocyte maturation rate at 18 h IVM in the presence or absence of etomoxir. **C.** MAPK14 and MAPK3/1 phosphorylation in CC after 18 h IVM in the presence or absence of etomoxir. Histograms correspond to mean ratio +/−SEM of 4 independent replicates. Mann–Whitney test was performed. Asterisks denote significant difference; *p < 0.05, **p < 0.01, ***p < 0.001.

As shown in Figure 6A, after 6 h of IVM in the presence of etomoxir oocytes had lower MAPK3/1 phosphorylation level as compared to control. After 18 h IVM, oocyte maturation rate was significantly lower in COCs treated with etomoxir than in control COCs matured without inhibitor (1.2% vs 52.1%) as shown in Figure 6B. Regarding signaling pathways, after 6 h IVM neither MAPK3/1 nor MAPK14 phosphorylation differed between treated and control COCs (not shown); nevertheless 30% increase of MAPK14 phosphorylation (p < 0.05), but not MAPK3/1 phosphorylation, was observed in response to etomoxir after 18 h IVM (Figure 6C).

## Discussion

### Expression of Tribbles genes in cumulus cells and their possible involvement in the regulation of oocyte maturation

*Tribbles* gene expression was shown to be tissue- and cell-specific [2,36]. Here we described for the first time the expression of *Tribbles* genes in CC and the enclosed oocytes in mice and bovine. In CC surrounding immature and preovulatory in vivo matured oocytes, *Tribbles* displayed similar pattern of variation in both mammalian species. This is likely due to the similarity of their kinase-like domains [37] and may indicate a functional conservation of each Tribble gene in CC of both species during oocyte maturation. *TRIB1* and *TRIB3* were up-regulated, whereas *TRIB2* was down-regulated during the preovulatory period in CC, and the same variation of *TRIB2* expression was shown in bovine granulosa cells after hCG-induced ovulation [12]. Expression of the three *Tribble*s genes in bovine CC was down-regulated by IVM serum-free culture of COCs. Such differences in expression between *in vivo* and *in vitro* could be explained by the significant differences of follicular fluid and IVM medium composition and also by contamination of expanded preovulatory cumulus with blood and plasma components originating from follicular fluid. Indeed, *Tribbles* transcripts have been detected in blood cells as reported in humans [38].

*Tribbles* regulate cell proliferation via different pathways, as reported for *TRIB1* in different cell types [39] and for *TRIB2* in tumors [40]. The overexpression of *TRIB2* was observed in cancer cells, where regulation of growth arrest is altered [40]. During maturation, CC do not actively proliferate, therefore the decrease of *TRIB2* and the increase of *TRIB1* and *TRIB3* levels in CC surrounding mature oocyte coincided with reduced proliferating activity of CC after maturation. When CC were cultured in vitro in the presence of 10% of foetal calf serum and IGF, they were actively proliferating. In these CC, *Tribbles* proteins were detected in both the cytoplasm and nucleus. This is consistent with the data obtained in human concerning TRIB1 in blood T-cells [41], for TRIB2 in LT7 cancer cells [42] and for TRIB3 in Muller cells [43]. In the cytoplasm, the Tribbles proteins direct some key signaling proteins to the proteasome, but they also modulate signaling, such as the MAPK pathway, or play a decoy kinase role [4]. Nuclear localization of TRIB1, TRIB2 and TRIB3 was more representative in proliferating CC during *in vitro* culture and much less obvious in non-dividing CC. In mitotic cells, TRIB2 and TRIB3 were more concentrated around the chromosomes and spindle. Moreover, TRIB2 was associated with interzona in anaphase and telophase dividing cells. These data also imply the roles of Tribbles in cell proliferation and mitosis in follicular cells, similarly to other cell types in compliance with previous studies [39].

Different expression variations of the *Tribbles* members in CC during oocyte maturation indicated that they may have different roles in this process. During IVM, *TRIB1* and *TRIB3* showed similar expression pattern with a high transient increase after 3 h and 6 h of IVM followed by a decrease to the basal level at the end of IVM. This peak of *TRIB1* and *TRIB3* expression in CC coincided with a period of oocyte germinal vesicle breakdown (GVBD, at 6 h IVM), suggesting that these genes may participate in the CC pathways regulating this process. Among those pathways, the importance of MAPK3/1 and MAPK14 signaling in the regulation of bovine oocyte maturation was previously highlighted [33,35,44]. Moreover, Tribbles also take part in these pathways in different cell types [31,32,45]. For instance, the increase of *TRIB3* expression in *HeLa* cervical carcinoma cells resulted in the inhibition of MAPK14, MAPK3/1 and MAPK8 (JNK) activities *via* binding to MAPK kinase [31]. In CC, MAPK14 phosphorylation increased at 6 h-10 h IVM, and then its decrease is concomitant with meiosis completion of bovine oocytes [44]. The variation of *TRIB1* and *TRIB3* expression at that time may be related to MAPK14 phosphorylation in CC and therefore to oocyte meiosis progression. Indeed, post-GVBD meiotic arrest related to GSK3 activity inhibition was associated with higher MAPK14 phosphorylation [44]. In our study, in the response to etomoxir, phosho-MAPK14 was increased and oocyte maturation was delayed as evidenced after 18 h IVM and this was associated with a decrease of MAPK3/1 phosphorylation in the oocytes at 6 h IVM. *TRIB1* and *TRIB3* expression was up-regulated and *TRIB2* was down-regulated in CC of treated COCs as compared to control. Moreover, expression of *Tribbles* genes in the oocyte and concentration of TRIB2 and TRIB3 proteins in germinal vesicle observed in this study, in bovine and mice oocytes, emphasized their possible involvement in the nuclear maturation of oocytes. Yamamoto et al. reported that *Trib1*-deficient female mice and Drosophila in adulthood are both infertile (unpublished data cited in [10]). Indeed no data on subfertility of *Trib2* and *Trib3* knock-out mice was reported. The eventual lack of one of these *Tribbles* (notably in *Trib2−/−o*r *Trib3−/−m*ice) may be compensated by another gene of *Tribbles* family (due to a high similarity between the *Tribbles* members) and thus to preserve female fertility.

### Alteration of Tribbles expression in cumulus cells and oocyte maturation delay in response to FAO inhibition during IVM

**Figure 7:**
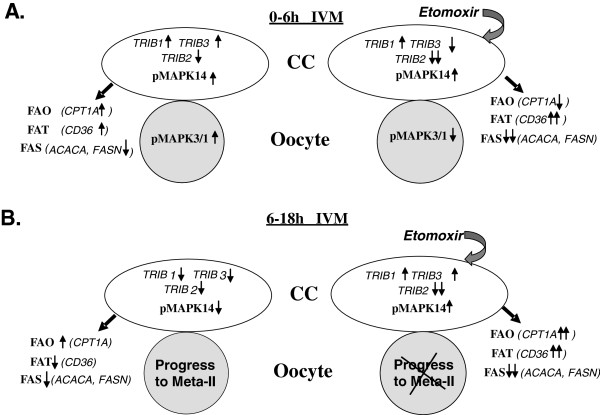
**Recapitulative scheme of the effect of etomoxir (150 μM) on oocyte maturation, expression of *Tribbles *** **and genes of FA oxidation (FAO), FA transport (FAT) and FA synthesis (FAS), and on MAPK signaling in oocyte and in CC at 6 h IVM (A) and 18 h IVM (B).**

The presence of the main factors of FA metabolism, such as fatty acid synthase FASN and active hormone sensitive lipase LIPE, was reported in bovine CC and in the oocyte in our previous study [19]. Also, expression of other genes related to lipogenesis and to FAO was detected in CC in different species [46,29] indicating that these cells can produce and metabolize FA. The present study demonstrates that expression of *Tribbles,* as well as FA metabolism-related genes, was significantly altered in response to FAO inhibitor etomoxir, in parallel to the enclosed oocyte maturation arrest that was observed, similar to other studies [24,29]. Different regulation of *Tribbles* genes in response to etomoxir in CC implies possible roles of *Tribbles* in FA metabolism. Here we reported that the three *Tribbles* were differently regulated in response to FAO inhibition at different times of IVM. Thus, at 6 h IVM, both *TRIB2* and *TRIB3* were reduced in expression in CC after etomoxir treatment than in control, and this coincided with decreased expression of FA oxidation, FA synthesis genes and increase of FA transport gene *CD36* in CC and with a lower level of MAPK3/1 phosphorylation in the enclosed oocyte (Figure 7A). Further expose of COCs to etomoxir (18 h IVM) led to significant reduction of oocyte meiosis progression accompanied by higher *TRIB1* and *TRIB3* and lower *TRIB2* expression level in CC (Figure 7B). Several recent studies have implicated the *Tribbles* family in lipid metabolism (reviewed in [47]). Thus, TRIB1 has been associated with disregulated triglycerides [48] and plasma cholesterol levels in humans [6]. *TRIB1* was described as a gene regulating hepatic lipogenesis and very low density lipoprotein production, and *Trib1* deficiency lead to accumulation of plasma triglycerides in mice [7]. Trib3 is involved in modulating lipid metabolism within adipose tissue in mice [23]. Also, TRIB3 suppressed promoter activity of the gene *PLIN2* encoding a protein associated with lipid droplets *via* a direct protein-protein interaction with PPARG in 3 T3-L1 adipocytes [49].

PPARG is one of the transcriptional factors regulating FA storage and glucose metabolism by activating genes that stimulate lipid uptake and adipogenesis by adipose cells [43]. Administration of PPARG agonist rosiglitazone during *in vitro* maturation reduced FAO in mice COCs and altered expression pattern of *Cpt1b, Cpt1c and Cpt2* genes [50]. We detected the expression of *PPARG* in bovine CC and this was shown to be affected by etomoxir during IVM (tend to increase, p < 0.1). Also, *CD36*, one of the target genes of PPARG was significantly up-regulated by etomoxir similar to *TRIB3*. TRIB3 was shown to bind PPARG and negatively regulate its transcriptional activity in differentiating adipocytes [49]. Thus, in follicular cells TRIB3 may be involved in lipid metabolism regulation *via* control of PPARG activity. The up-regulation of *CD36* in etomoxir-treated CC may be explained by the possibly increased amounts of saturated FA (such as palmitic acid) as a result of etomoxir inhibition of CPT1 activity which blocks FA entry into mitochondria. This is in line with the studies that showed that palmitic acid induced a rise in *CD36* mRNA in bovine mammary cell culture [51] or in chicken skeletal muscle [52]. Moreover, *CD36* is a mediator of inflammation, including that which responds to excess fat supply [53]. Ovulation is also related to inflammatory process, and in mice CC *Cd36* expression increased 4 h after administration of an ovulatory dose of gonadotropins to COCs in vitro [54]. In our experiments, *TRIB2* variations were opposite to those of *CD36*. The inverse correlation between inflammatory response and *TRIB2* expression was reported in human primary monocytes, highlighting TRIB2 as an important controller linking lipid levels to inflammatory response in these cells [55]. Significant down-regulation of *TRIB2* in bovine CC during the preovulatory period *in vivo* and after IVM may infer a similar role for this gene in bovine COCs.

*TRIB3* up-regulation during IVM may thus prevent CC from accumulating lipids during *in vitro* culture as was shown in proliferating preadipocytes where *TRIB3* was up-regulated in response to α-tocopherol phosphate, whereas in differentiated adipocytes *TRIB3* was repressed [9]. Increase of *CPTA1* and decrease of *ACACA* and *FASN* expression in etomoxir-treated CC coincided with increase of *TRIB3* expression. In fact, up-regulation of *CPT1A* in CC during IVM suggests the increasing requirement for lipid utilization and activated FAO in these cells, which was more pronounced after etomoxir treatment. Also, TRIB3 binds to ACC and to the E3 ubiquitin ligase COP1 through its COP1 site leading to the degradation of ACC by the proteasome in mice adipose tissue [23]. According to our experiments, TRIB3 might be thus also involved in a regulation of lipogenesis in CC during IVM. Therefore, under the treatment with etomoxir, TRIB3 could act as a nutrient sensor and prevent cells from undergoing apoptosis as described in prostate cancer cells [8]. Indeed, etomoxir was reported to markedly reduce cellular ATP levels in human glioblastoma SF188 cells [56] as well as in bovine COCs [29], thus increasing the AMP/ATP ratio, and consequently activating AMPK. AMPK phosphorylation leads to the inhibition of FA synthesis, through Acetyl CoA carboxylase (ACC phosphorylation/inhibition), reducing Malonyl CoA availability and consequently increasing CPT1 activity [for review [57]]. In fact, using inhibition of FAO by etomoxir and malonyl CoA, two inhibitors of CPT1, FAO in COCs was shown to be necessary for meiosis resumption in mice [24,28]. Moreover, treatment of COCs with etomoxir during IVM blocked meiosis progression also in bovine and porcine species [29]. Because Tribbles proteins were also shown in oocytes, where the importance of FA metabolism is crucial for oocyte maturation and developmental competence [19,24,25,27,28], we suggest their possible involvement in the regulation of related metabolic processes that finally lead to correct meiosis progression. Nevertheless, involvement of *Tribbles* and other FA-metabolism related genes in oocyte maturation may differ in mice and bovine COCs because these species are quite different regarding oocyte lipid metabolism. In fact, mice oocytes have significantly lower levels of intracellular lipids than bovine oocytes and they are therefore much less sensitive to FAO inhibition *in vitro*[29]: In fact, in response to etomoxir mice oocytes can rapidly adapt their metabolism to another energy sources in contrast to cow and pig [29]. Also, mice and bovine oocyte maturation is oppositely regulated by AMPK in vitro: pharmacological AMPK activators triggered meiotic resumption in mice [24,26,58] whereas in bovine and porcine the same treatment inhibited meiotic maturation [59,60].

In conclusion, expression of *Tribbles* family genes in the cumulus-oocyte complex revealed their possible involvement in the regulation of cumulus functioning both during proliferation and during meiosis of the enclosed oocytes. *Tribbles* may also be involved in lipid metabolism of these cells. Other investigations need to be pursued to fully understand the precise roles of each of the *Tribbles* genes products in cumulus cells-oocyte dialog during oocyte maturation.

## Competing interests

The authors declare that there is no conflict of interest that could be perceived as prejudicing the impartiality of the reported research.

## Authors’ contributions

DB carried out gene expression analysis in bovine CC, participated in IVM and immunofluorescence analysis, performed statistical analysis and drafted the manuscript. FC carried out gene expression analysis in mice and participated in draft of the manuscript. SE participated in design of the study; performed primers’ design and validation and participated in writing of the manuscript. AD performed WB analysis; LSL and MC carried out IVM of bovine COCs and participated in expression analysis; VM carried out analysis on mice COCs and helped to draft the manuscript. SU conceived the study, participated in IVM, immunofluorescence analysis, figure design and writing of the manuscript. All authors read and approved the final manuscript.

## Supplementary Material

Additional file 1: Table S1 List of primers used for RT-qPCR analysis. Click here for file

Additional file 2: Figure S1 Immunofluorescence detection of TRIB2 and TRIB3 proteins in mice oocyte-cumulus complexes. First column represents typical merge confocal images of Tribbles and Hoechst labeling of entire cumulus-oocyte complex (COC). White arrows indicate germinal vesicle. Antibodies against rh TRIB2 and TRIB3 proteins and Alexa488-coupled secondary antibodies were used to detect specific labeling. Rabbit IgG were used as negative control. Bars = 50 μM. Click here for file

Additional file 3: Figure S2 Morphological estimation of viability of cumulus cells inside of cumulus-oocyte complex using Live/Dead Viability assay after 18 h of in vitro culture of COCs in the presence (Etomoxir 150 μM) or absence (Control) of fatty acid oxidation inhibitor etomoxir. Green – live cells; red- dead cells. Click here for file
