# Supplementary material for: Tribbles expression in cumulus cells is related to oocyte maturation and fatty acid metabolism
Source: J Ovarian Res. 2014 Apr 26;7:44. doi: 10.1186/1757-2215-7-44 (PMC4022380; doi:10.1186/1757-2215-7-44)
Supplement: Additional file 1: Table S1 — List of primers used for RT-qPCR analysis. [file 1757-2215-7-44-S1.doc]

**Table 1.** List of primers used for RT-qPCR analysis in cow.

| **Gene ID** | **Primer** | **Sequence (5’-3’)** | **Accession Number** | **Amplicon size (bp)** | **Gene description** |
| --- | --- | --- | --- | --- | --- |
| *ACACA* | forward | TGCTTCCCATTTGCCATC | NM_174224 | 188 | Acetyl coenzyme A carboxylase |
|  | reverse | CTGCCATCCTCACGACCT |  |  |  |
| *CD36* | forward | GCATTCTGAAAGTGCGTTGA | BC103112.1 | 181 | CD36 molecule (thrombospondin receptor) |
|  | reverse | CGGGTCTGATGAAAGTGGTT |  |  |  |
| *CPT1A* | forward | TCCTGGTGGGCTACCAATTA | FJ415874 | 181 | Carnitine palmitoyltransferase 1A |
|  | reverse | TGCGTCTGTAAAGCAGGATG |  |  |  |
| *FASN* | forward | CACTCCATCCTCGCTCTCC | AY343889 | 181 | Fatty acid synthase |
|  | reverse | GCCTGTCATCATCTGTCACC |  |  |  |
| *PPARG* | forward | CCCTGGCAAAGCATTTGTAT | Y12419/Y12420 | 222 | Peroxisome proliferator activated receptor gamma |
|  | reverse | ACTGACACCCCTGGAAGATG |  |  |  |
| *SCD1* | forward | CTGGCTGGTGAATAGTGCTG | NM_173959 | 129 | Stearoyl-coenzyme A desaturase-1 |
|  | reverse | AAGGTGTGGTGGTAGTTGTGG |  |  |  |
| *TRIB1* | forward | TGGCACAAAAACAGGCAGTCACCT | NM_001101105.2 | 359 | Tribbles homolog 1 (Drosophila) |
|  | reverse | CGAAGCCCCAGCAAACCCAGA |  |  |  |
| *TRIB2* | forward | GTGGCATGTAGTGCAGACC | NM_178317.3 | 300 | Tribbles homolog 2 (Drosophila) |
|  | reverse | ACAGGACAAAGCACCAGAG |  |  |  |
| *TRIB3* | forward | TGCAGATGAGCTCGTGCCCC | NM_001076103.1 | 305 | Tribbles homolog 3 (Drosophila) |
|  | reverse | AGGCCTGGAGCCTTTGGCAC |  |  |  |
| *RPS9* | forward | GGAGACCCTTCGAGAAGTCC | NM_0011011522 | 180 | Ribosomal protein S19 |
|  | reverse | GGGCATTACCTTCGAACAGA |  |  |  |
| *RPL19* | forward | AATCGCCAATGCCAACTC | BC102223 | 156 | Ribosomal protein L19 |
|  | reverse | CCCTTTCGCTTACCTATACC |  |  |  |

List of primers used for RT-qPCR analysis in mouse.

| **Gene ID** | **Primer** | **Sequence (5’-3’)** | **Accession Number** | **Amplicon size (bp)** | **Gene description** |
| --- | --- | --- | --- | --- | --- |
| *Trib1* | forward | CCTCGAATATGGCAGCATTT | NM_144549 | 100 | Tribbles homolog 1 (Drosophila) |
|  | reverse | CGAGTCTCCTCACCCTTGTC |  |  |  |
| *Trib2* | forward | TTGGAACAGACCAACCACCT | NM_144551.5 | 98 | Tribbles homolog 2 (Drosophila) |
|  | reverse | TTTAGCACCCAGGTTTCAGG |  |  |  |
| *Trib3* | forward | GGAACCTTCAGAGCGACTTG | NM_175093.2 | 101 | Tribbles homolog 3 (Drosophila) |
|  | reverse | TCTCCCTTCGGTCAGACTGT |  |  |  |
| *Rplp0* | forward | TGCCACACTCCATCATCAAT | NM_007475 | 96 | Ribosomal protein large P0 |
|  | reverse | AGGAAGGCCTTGACCTTTTC |  |  |  |
| *Rn18S* | forward | TAGAGGGACAAGTGGCGTTC | NR_003278.3 | 103 | 18S ribosomal RNA |
|  | reverse | CGCTGAGCCAGTCAGTGTAG |  |  |  |
