# Supplementary figures and images for: Tribbles expression in cumulus cells is related to oocyte maturation and fatty acid metabolism
Source: J Ovarian Res. 2014 Apr 26;7:44. doi: 10.1186/1757-2215-7-44 (PMC4022380; doi:10.1186/1757-2215-7-44)

## Slide 1
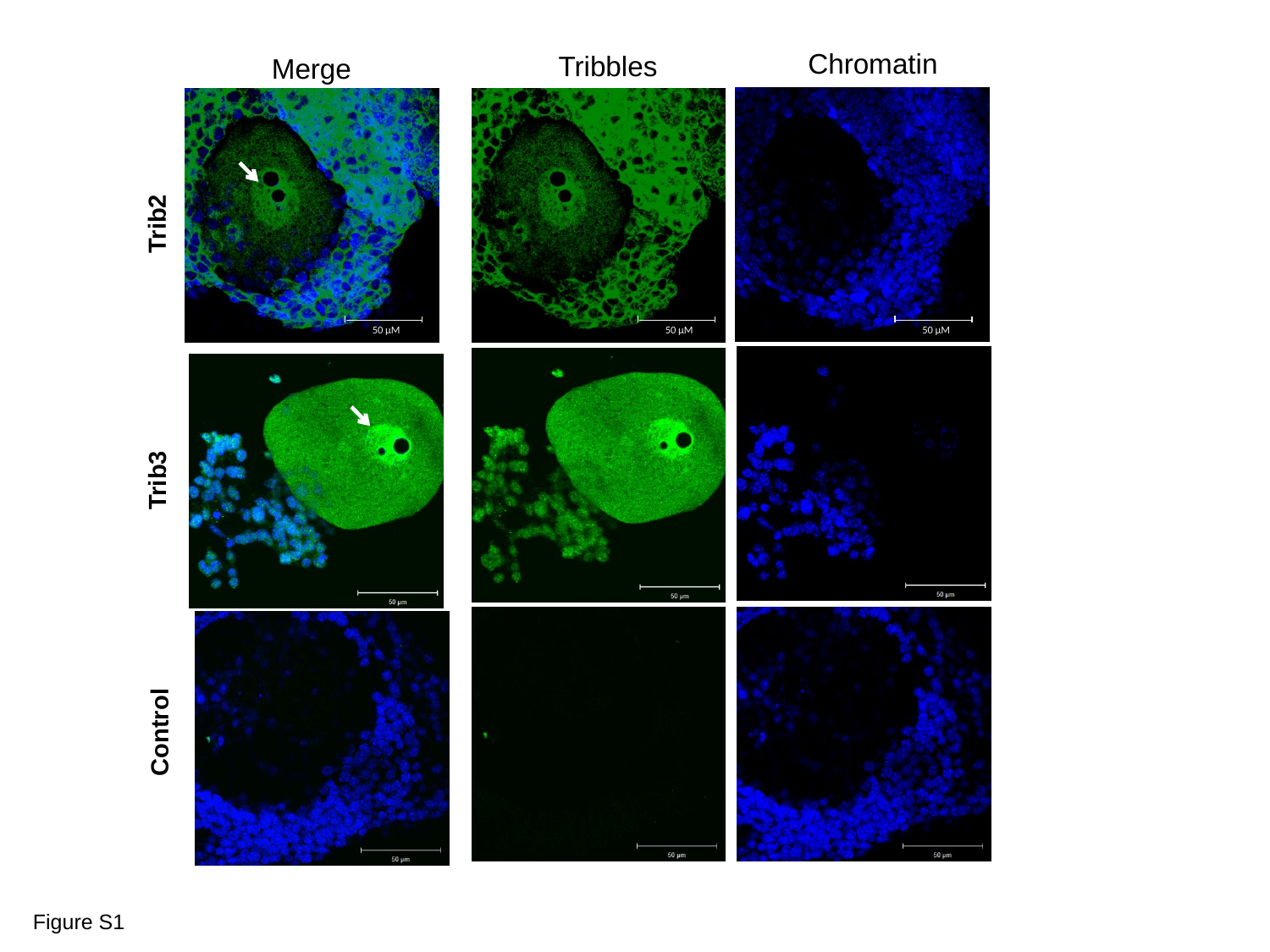

Chromatin
Tribbles
Merge
Alexa488
Hoechst
Merge
Trib2
50 µM
50 µM
50 µM
Trib3
Control
Figure S1

Supplement: Additional file 2: Figure S1 — Immunofluorescence detection of TRIB2 and TRIB3 proteins in mice oocyte-cumulus complexes. First column represents typical merge confocal images of Tribbles and Hoechst labeling of entire cumulus-oocyte complex (COC). White arrows indicate germinal vesicle. Antibodies against rh TRIB2 and TRIB3 proteins and Alexa488-coupled secondary antibodies were used to detect specific labeling. Rabbit IgG were used as negative control. Bars = 50 μM. [file 1757-2215-7-44-S2.pptx]

## Slide 1
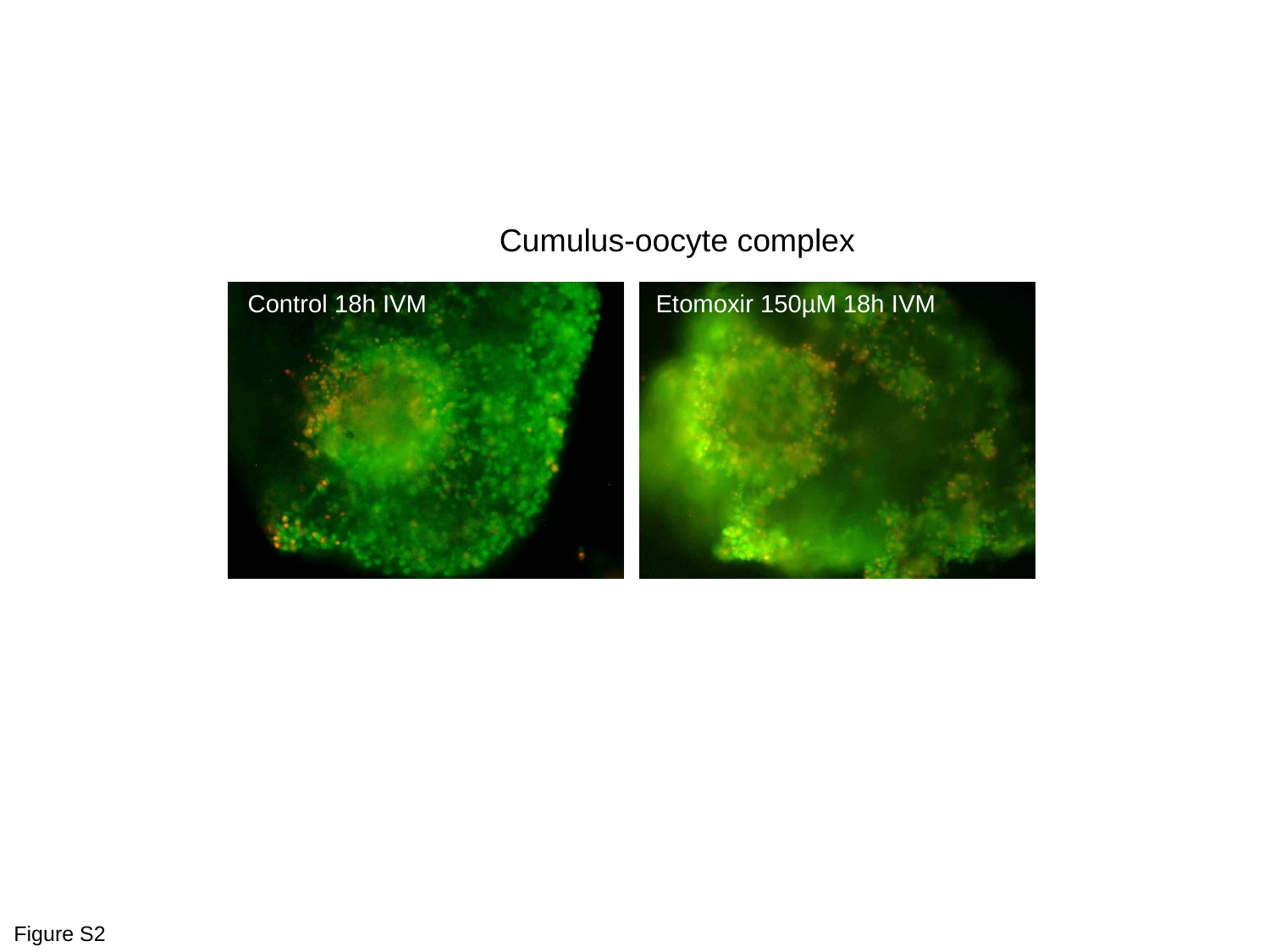

Cumulus-oocyte complex
Etomoxir 150µM 18h IVM
Control 18h IVM
Figure S2

Supplement: Additional file 3: Figure S2 — Morphological estimation of viability of cumulus cells inside of cumulus-oocyte complex using Live/Dead Viability assay after 18 h of in vitro culture of COCs in the presence (Etomoxir 150 μM) or absence (Control) of fatty acid oxidation inhibitor etomoxir. Green – live cells; red- dead cells. [file 1757-2215-7-44-S3.pptx]
